# The influence of perceived parenting styles on socio-emotional development from pre-puberty into puberty

**DOI:** 10.1007/s00787-017-1016-9

**Published:** 2017-06-19

**Authors:** Min Yee Ong, Janna Eilander, Seang Mei Saw, Yuhuan Xie, Michael J. Meaney, Birit F. P. Broekman

**Affiliations:** 1 Singapore Institute for Clinical Sciences, Agency for Science, Technology and Research, Singapore, Singapore; 2 Department of Child and Family Studies, Faculty of Social and Behavioural Sciences, Leiden University, Leiden, The Netherlands; 3 Department of Psychiatry, VU Medical Centre, Amsterdam, The Netherlands; 4 Department of Psychiatry, Queen’s University, Kingston, ON Canada; 5 Sackler Program for Epigenetics and Psychobiology, Ludmer Centre for Neuroinformatics and Mental Health, Department of Psychiatry, McGill University, Montreal, Canada; 6 Brenner Centre for Molecular Medicine, 30 Medical Drive, 117609 Singapore, Singapore

**Keywords:** Parenting, Child development, Behavioural problems, Socio-emotional development, Adolescence

## Abstract

The relative impact of parenting on socio-emotional development of children has rarely been examined in a longitudinal context. This study examined the association between perceived parenting styles and socio-emotional functioning from childhood to adolescence. We hypothesized that optimal parenting associated with improvement in socio-emotional functioning from childhood into early adulthood, especially for those with more behavioral problems in childhood. Children between ages 7 and 9 years were recruited for the Singapore Cohort Study of Risk Factors for Myopia (SCORM). Nine years later, 700 out of 1052 subjects were followed up (67%). During childhood, parents completed the Child Behavior Checklist (CBCL), while young adults completed the Youth Self-Report (YSR) and Parental Bonding Instrument (PBI). Perceived optimal parental care resulted in less internalizing and externalizing problems in early adulthood in comparison to non-optimal parental care styles. Perceived optimal paternal parenting, but not maternal parenting, in interaction with childhood externalizing problems predicted externalizing symptoms in early adulthood. No significant interactions were found between perceived parenting styles and internalizing problems. In conclusion, perceived parental care associates with the quality of socio-emotional development, while optimal parenting by the father is especially important for children with more externalizing problems in childhood.

## Introduction

The onset of most common mental disorders is before adulthood [1, 2]. The early onset of psychopathology implies a formidable cost for both the individual and society. The peripubertal peak in the onset of mental disorders underscores the importance of early interventions targeting at-risk children [3]. The ability to effectively identify children at risk is essential for the design and delivery of prevention programs [4–6]. These considerations suggest that it is critical to understand how socio-emotional functioning changes over development and to identify the course of this variation.

Emotional and behavioral dysregulation is an important predictor of mental disorders later in life [7]. However, the association between measures of emotional and behavioral problems in childhood and later psychopathology, while statistically significant, shows variation over time with evidence for a considerable portion of children exhibiting emotional or behavioral difficulties in early life, but without evidence of psychopathology at later ages [8, 9]. It is thus important to understand the sources of variation in emotions and behavior and to identify factors that moderate changes over time.

One of the most important factors that influence socio-emotional development is parenting [3, 10–14]. Positive forms of parenting are beneficial for cognitive and social development, while negative parenting such as punishment and low warmth are associated with disruptive behavior in children and increased risk for psychopathology [11, 15]. These effects are apparent in studies that used self-reports of perceived parenting styles using measures such as the Parental Bonding Instrument (PBI) [16]. For example, PBI scores characterized by low care and high overprotection predict psychopathology such as depression, anxiety disorders, and eating disorders [16–20].

Studies of the relationship between perceived parenting and psychopathology are most commonly explored with cross-sectional design and often conducted in adult samples [21–23]. The design of such studies does not permit an analysis of the potential role of parenting as a moderator of the relationship between early risk and later outcomes. In addition, most studies focus on clinical samples, or on groups with low socio-emotional status [24]. Hence, these samples do not inform about the extent of perceived parenting effects on socio-emotional development in a general population.

The aim of this study is to use a longitudinal design to examine whether parenting, as measured with the PBI, is a moderator of the relation between early socio-emotional functioning and later mental health. We hypothesize that perceived optimal parenting, characterized by high warmth and low control, in interaction with behavioral and emotional profiles in middle childhood, will have a positive effect on the socio-emotional development over time and lead to less reported symptoms of psychopathology during late adolescence/early adulthood. Moreover, since the effects of intervention programs that target parental care are often greatest among children with negative temperament [25], we predicted that the effect of optimal parenting would be most apparent among children with increased emotional and behavioral problems in childhood.

## Methods

### Participants

The current study sample was derived from the SCORM that recruited a total of 1979 children out of 2913 Asian children (participation rate: 67.9%) from three normal stream schools in Singapore between November 1999 and May 2001. The majority of the children in Singapore attend normal stream schools, while children with intellectual disability (IQ <70) often attend ‘special’ schools [26]. Children with serious chronic medical conditions (e.g., heart disorders, cancer, and chronic eye conditions) were excluded (*n* = 94).

### Measures of internalizing and externalizing traits

The Child Behavior Checklist (CBCL) 4–18 parental report and the Youth Self-Report (YSR) are both tests of emotional and behavioral difficulties for, respectively, parents of children between 4 and 18 years old (CBCL), and for adolescents between 11 and 18 years of age (YSR). The CBCL consists of 118 items and the YSR 112 items, both assessed with a three-point Likert scale, with eight subscales, two syndrome groups, and a total problem score each. The two syndrome groups are internalizing and externalizing scale scores. The internalizing scale score is a grouping of social withdrawal, somatic complaints and anxiety/depression items, whereas the externalizing score is a grouping of delinquent behavior and aggressive behavior items [27]. We used raw scores for both the CBCL and YSR [28]. The CBCL and YSR have good construct validity and acceptable test–retest reliability coefficients among the subscales. Cronbach’s *α* values range from .62 to .92 [27].

### Measurement of parenting styles

The PBI is a widely used battery for assessing parenting behaviors [29]. It is a self-report questionnaire that asks participants retrospectively about the perceived parenting styles of both parents during the first 16 years of their life. Participants complete sections for mother and father separately. PBI measures perceived levels of care and overprotection/control. It consists of 25 items, 12 ‘care’ items and 13 ‘overprotection’ items [7]. The reliability and validity of the PBI have been found to be satisfactory [29].

### Measurements of demographic and socioeconomic data

Parents completed a questionnaire, in English or Chinese, on demographic information inclusive socioeconomic indicators at the baseline visit. Ethnicity was determined based on the father’s reported ethnicity [30]. Socioeconomic status was assessed on the basis of maternal education, a frequently used index in studies with children.

### Procedures

Before commencing study, all parents provided written informed consent while children provided written assent. At baseline, the children were in grades one to three (7–9 years old). At that time the children were assessed with an IQ test (Raven Standard Progressive Matrices), parents provided information on demographics and filled out the English version of the CBCL. Of the initial SCORM sample of 1979 children, only 1336 completed the CBCL, because one of the three schools did not participate in completing the CBCL. Subsequently, participants were followed up 9 years after baseline. During follow-up, between the ages of 16 and 21, a package consisting of a consent form on the follow-up study and a set of self-report questionnaires including YSR and PBI in English were mailed to 1052 participants. After giving consent, the participants were to complete and mail back the set of questionnaires to the study team. Seven hundred of the 1052 participants gave consent for the follow-up study (response rate 66.5%). Among those 700 participants, 460 had complete data on the CBCL, YSR and PBI; hence, they were included for analyses in this study. The Singapore Eye Research Institute Ethics Committee approved the initial and follow-up study and the tenets of the Declaration of Helsinki were observed.

### Statistics

To appreciate parenting effects, we were especially interested in those children with discrepancies between their CBCL and YSR scores. This includes one group with high CBCL score at age 7–9 years, but low YSR score at young adulthood, and another group with an initial low score on the CBCL when young, but high YSR score at later stage in life.

CBCL and YSR raw scores were used and adjusted for gender and age. Differences in ages, gender, ethnicity, socioeconomic status, and IQ between the included and excluded samples were analyzed by using parametric tests if normality and homogeneity assumptions were satisfied; otherwise, the non-parametric Mann–Whitney *U* test was used. Categorical data were analyzed using the Chi-squared test.

The association between scores on the CBCL and YSR in interaction with parenting styles was analyzed as categorical variables in general linear models, where CBCL and YSR were divided into four groups: low scores, low average scores, high average scores and high scores. For parenting styles, comparisons were made between optimal versus “non-optimal parenting” (which groups affectionate constraint, affectionless control, and neglectful parenting) as well as optimal versus affectionless control, neglectful, and affectionate constraint parenting separately [31].

All outliers above 3 standard deviations were removed. All statistical procedures were performed with the Statistical Package for the Social Sciences (SPSS) 16.0 (SPSS Inc., Chicago, IL, USA) and *p* values were two-tailed and considered statistically significant when the values were below .05.

## Results

Of the 460 participants with complete data on the CBCL, YSR and PBI, 4 were older than 21 years and 8 with missing age; hence, they were excluded for analyses in this study. Based on scatter plots, three outliers (i.e., >3 SD away) were removed. Thus, data from 445 participants with complete data (192 boys and 253 girls) were included in our final analyses.

The age ranged from 6 to 10 years with a mean of 7.8 years (SD = 0.83) at the time of baseline (CBCL) and 16 to 21 years with a mean of 18.4 years (SD = 1.32) at the second assessment that included the YSR and PBI. The mean score for internalizing problems on the CBCL raw score was 7.68 (SD = 7.53, range 0–58) and 7.71(SD = 7.06, range 0–65) for externalizing problems. The mean score for internalizing problems on the YSR raw score was 13.0 (SD = 8.4, range 0–48) and 10.5 (SD = 6.8, range 0–33) for externalizing problems. There was a high correlation between the CBCL and YSR total raw scores as well as sub-scores (*p* < .001). No main effects on maternal education or IQ were found. Hence, these variables were not included in subsequent analyses.

Comparison on group characteristics were conducted for those included for analysis (*n* = 445) and those excluded from analysis (*n* = 255) in the group of 700 children who were followed up with questionnaires returned. Chi-squared analysis was conducted to compare group differences in gender, race, income, and housing, while non-parametric Mann–Whitney *U* test was conducted to compare group differences in age and IQ scores, as they were not normally distributed in our sample (with more children having higher IQs and more children being 7 instead of 8 or 9 years old at baseline). No significant difference was found between the groups (see Table 1). We also explored if there were differences in PBI and CBCL between the two groups using Chi-square analysis for PBI groups and independent *t* test for CBCL. Likewise, no significant difference was found between the groups for PBI and CBCL (see Tables 2, 3 for PBI and CBCL analysis, respectively).

**Table 1:** Comparison on demographic variables of children included for analysis (*N* = 445) and excluded from analysis (*N* = 255) from the group with returned questionnaires at follow-up visit

	Remained in follow-up visit (*N* = 700)		
	Included for analysis (*N* = 445)	Excluded for analysis (*N* = 255)	*X* (*p* value)
Gender			0.643 (.423)
Boys	192	118	
Girls	253	137	
Race			6.09 (.193)
Chinese	347	198	
Malay	65	40	
Indian	30	13	
Others	2	0	
Missing	1	4	
Income			5.94 (.051)
$2000 or less/mth	121	89	
>$20,000–$50,000/mth	212	115	
>$5000/mth	106	46	
Missing	6	5	
Housing			4.77 (.190)
1–3 Room	49	41	
4 Room and above	345	182	
Private housing	25	18	
Others	26	13	
Missing	0	1	
Father education level			4.14 (.388)
No formal education	11	9	
Elementary school	100	66	
High school	197	116	
Pre-university/diploma	76	40	
University	61	24	
Mother education level			6.92 (.140)
No formal education	13	10	
Elementary school	108	77	
High school	228	115	
Pre-university/diploma	69	40	
University	37	13	
			*U* (*p* value)
Age (at first visit) Mean (SD)	7.80 (0.83)	7.92 (0.88)	52,919.5 (.113)
IQ (at first visit) Mean (SD)	116.08 (10.7)	114.25 (11.66)	38,713 (.068)

**Table 2:** Comparison on PBI score distribution according to bonding type, means and standard deviation on perceived level of care and control by parents in children included for analysis (*N* = 445) and excluded from analysis (*N* = 255)

Remained in follow-up visit (*N* = 700)			
	Included for analysis (*N* = 445)	Excluded for analysis (*N* = 255)	*X* (*p* value)
Maternal			
Bonding type (%)			
Optimal	30.8	22.0	
Neglectful	18.0	14.5	
Affectionate constraint	17.8	11.0	
Affectionless Control	28.8	24.7	
Missing	4.7	27.8	
Maternal care [Mean (SD)]	26.20 (6.16)	26.33 (6.30)	28.6 (.486)
Maternal control [Mean (SD)]	13.30 (6.32)	13.56 (6.90)	32.0 (.466)
Paternal			
Bonding type (%)			
Optimal	27.2	18.4	
Neglectful	20.0	12.9	
Affectionate constraint	17.3	10.2	
Affectionless control	27.0	23.1	
Missing	8.5	35.3	
Paternal care [Mean (SD)]	23.16 (6.73)	22.66 (7.33)	38.5 (.235)
Paternal control [Mean (SD)]	11.84 (6.35)	12.43 (6.70)	35.0 (.284)

**Table 3:** Comparison on CBCL score [means (SD)] between groups of children included for analysis (*N* = 445) and excluded from analysis (*N* = 255)

Remained in follow-up visit (*N* = 700)			
CBCL Raw Score	Included for analysis (*N* = 445)	Excluded for analysis (*N* = 255)	*t* (*p* value)
Internalizing	7.68 (7.53)	7.30 (6.77)	−.444 (.657)
Externalizing	7.71 (7.06)	7.12 (6.03)	−.742 (.458)
Total	27.24 (22.46)	25.72 (20.01)	−.604 (.546)

### Perceived parenting styles in Singapore youths

The scores of the PBI showed that the majority of mothers and fathers were perceived by the participants to demonstrate optimal parenting styles, followed by affectionless controlled parenting styles. Using paired sample *t* test; significant differences on the PBI subscales “Care” and “Control” were found between Singaporean mothers and Singaporean fathers (see Table 2). Singaporean mothers scored higher on both care [*t*(399) = 9.81, *p* < .001] and control [*t*(400) = 5.44, *p* < .001] when compared with fathers.

### Effects of parenting styles on YSR

General linear model of CBCL categorical scores based on quartiles (low scores/below average/above average and high scores) in interaction with parenting styles on YSR were analyzed. The main effects showed that scores for participants reporting optimal maternal care and paternal care were significantly lower than for participants reporting “non-optimal care” (combination of all not-optimal parenting styles, namely “affectionless control”, “neglectful”, and “affectionate constraint”) on the YSR internalizing, externalizing and total score (see Table 4), When comparing optimal parenting with “affectionless control”, “neglectful”, and “affectionate constraint” parenting separately, all main effects were significant for all three YSR scales except for paternal affectionate constraint parenting.

**Table 4:** Main effects of perceived optimal parenting (*F* value) with other parenting style on YSR internalizing, externalizing and total score

	Mother	Father
YSR internalizing	22.7***	15.0***
Optimal	(*n* = 131)	(*n* = 117)
Non-optimal	(*n* = 272)	(*n* = 265)
	24.1***	16.9***
Optimal	(*n* = 131)	(*n* = 117)
Affectionless control	(*n* = 121)	(*n* = 112)
	1.94	0.53
Optimal	(*n* = 131)	(*n* = 117)
Affectionate constraint	(*n* = 78)	(*n* = 72)
	16.7***	11.6**
Optimal	(*n* = 131)	(*n* = 117)
Neglectful	(*n* = 73)	(*n* = 81)
YSR externalizing	17.1***	14.4***
Optimal	(*n* = 134)	(*n* = 117)
Non-optimal	(*n* = 276)	(*n* = 275)
	13.9***	12.6***
Optimal	(*n* = 134)	(*n* = 117)
Affectionless control	(*n* = 123)	(*n* = 114)
	2.60	4.10*
Optimal	(*n* = 134)	(*n* = 117)
Affectionate constraint	(*n* = 74)	(*n* = 74)
	16.8***	10.9**
Optimal	(*n* = 134)	(*n* = 117)
Neglectful	(*n* = 79)	(*n* = 87)
YSR total	23.6***	15.8***
Optimal	(*n* = 125)	(*n* = 109)
Non-optimal	(*n* = 247)	(*n* = 241)
	19.40***	18.2***
Optimal	(*n* = 125)	(*n* = 109)
Affectionless control	(*n* = 108)	(*n* = 98)
	5.82*	3.42
Optimal	(*n* = 125)	(*n* = 109)
Affectionate constraint	(*n* = 72)	(*n* = 67)
	17.6***	9.60**
Optimal	(*n* = 125)	(*n* = 109)
Neglectful	(*n* = 67)	(*n* = 76)

*** *p* < .001, ** *p* < .01, * *p* < .05

### Interactions between CBCL and perceived parenting styles

**Fig. 1:**
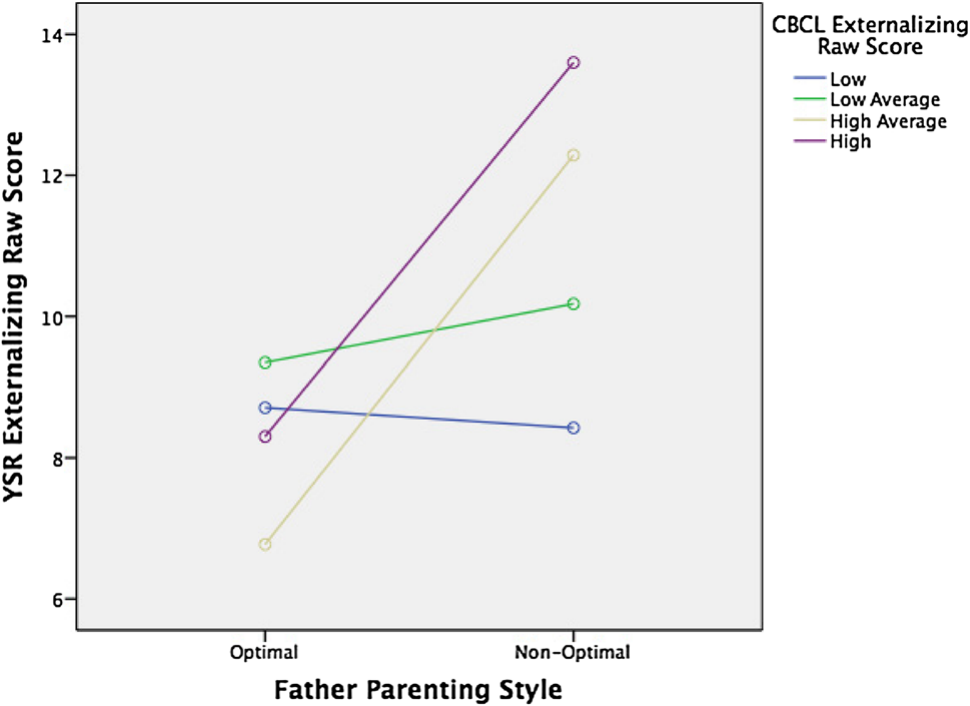
Interaction of paternal optimal parenting style (*n* = 117) vs. paternal non-optimal parenting style (*n* = 275) with CBCL externalizing raw scores (in quartiles) on YSR externalizing raw scores, *F*(3, 374) = 4.08*, p* = .007

**Fig. 2:**
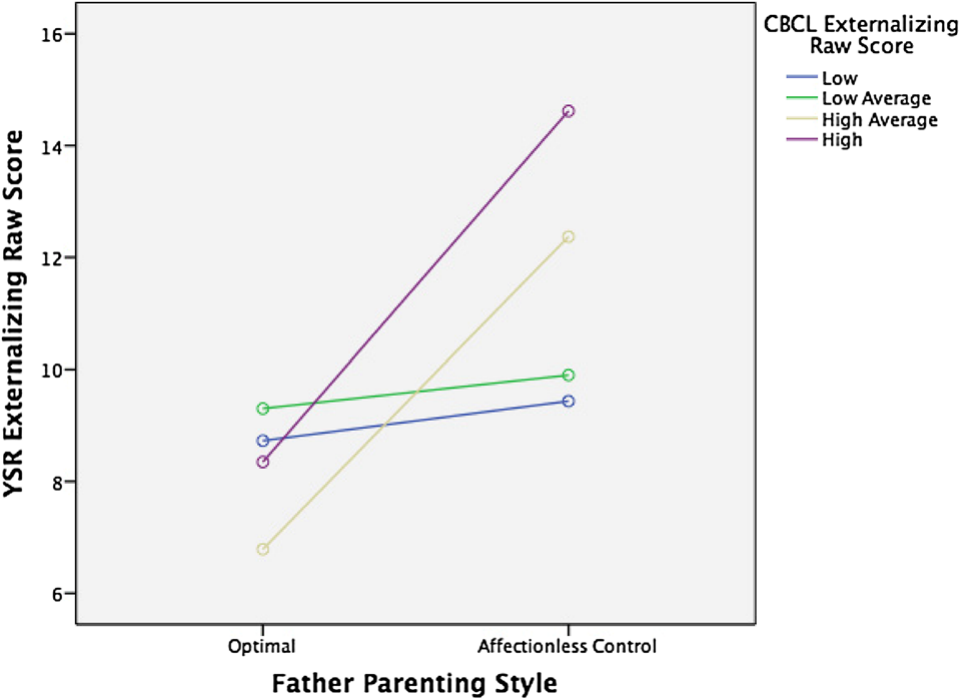
Interaction of paternal optimal parenting style (*n* = 117) vs. paternal affectionless control parenting style (*n* = 114) with CBCL externalizing raw scores (in quartiles) on YSR externalizing raw scores, *F*(3, 213) = 2.87*, p* = .037

**Fig. 3:**
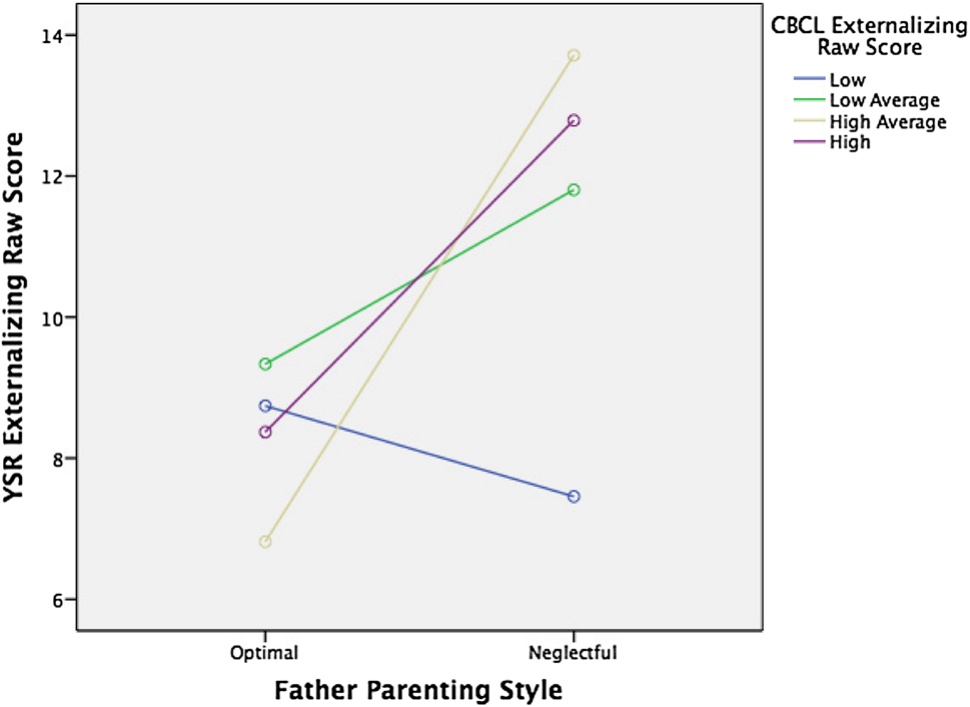
Interaction of paternal optimal parenting style (*n* = 117) vs. paternal neglectful parenting style (*n* = 87) with CBCL externalizing raw scores (in quartiles) on YSR externalizing raw scores, *F*(3, 186 = 3.50*, p* = .017

**Fig. 4:**
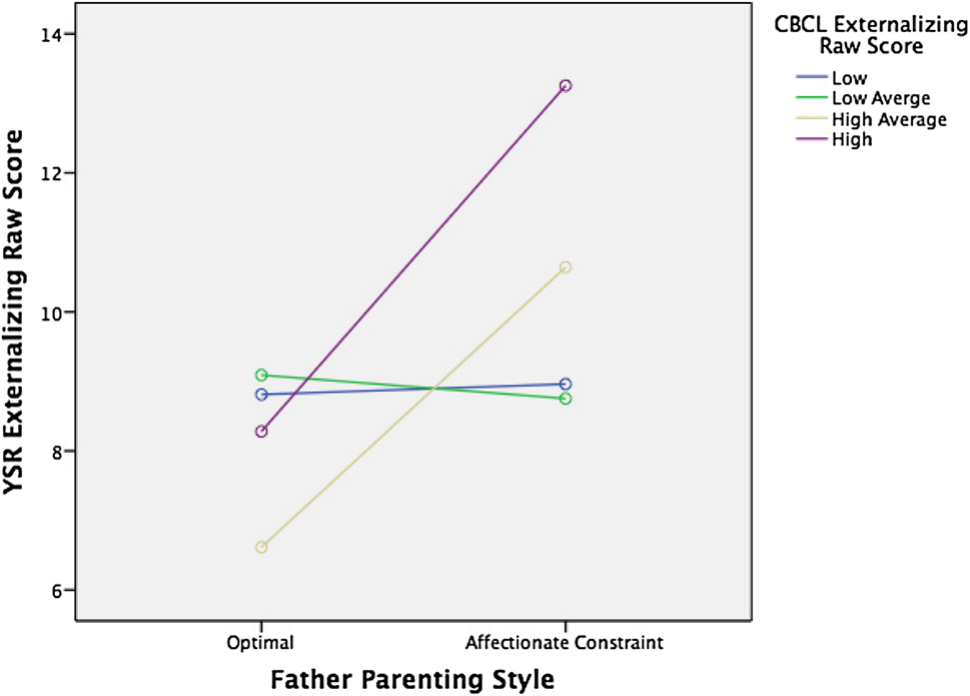
Interaction of paternal optimal parenting style (*n* = 117) vs. paternal affectionate constraint parenting style (*n* = 74) with CBCL externalizing raw scores (in quartiles) on YSR externalizing raw scores, *F*(3, 173) = 1.79*, p* = .15

No significant interaction was found between internalizing traits of childhood (CBCL) and parenting styles (of mother or father) on internalizing traits in adolescence/young adulthood (YSR). However, significant interactions were found between paternal parenting styles and externalizing traits in childhood (CBCL) and externalizing traits in adolescence/young adulthood (YSR). More specifically, a significant interaction was found between optimal parenting of the father and externalizing traits in childhood (CBCL) and externalizing traits in adolescence/young adulthood (YSR), *F*(3, 374) = 4.08, *p* = .007 (See Fig. 1). Also, significant interactions were found between paternal optimal parenting versus paternal affectionless control parenting [*F*(3, 213) = 2.87, *p* = .037] (See Fig. 2), and versus parental neglectful parenting and CBCL externalizing traits on YSR externalizing traits [*F*(3, 186) = 3.50, *p* = .017] (See Fig. 3), but only not versus affectionate constraint [*F*(3, 173) = 1.79, *p* = .15] (see Fig. 4) and externalizing traits in childhood on externalizing traits in young adulthood. In other words, and as shown in the figures, optimal care of the father lowers the risk of developing externalizing problems in youth for those children who had already higher externalizing scores in their childhood. Interestingly no interaction was found between any of the maternal parenting styles with internalizing or externalizing traits of the child (see Table 5).

**Table 5:** Interactions of perceived parenting style by fathers and mothers (optimal versus other parenting style) and CBCL internalizing, externalizing and total score on YSR internalizing, externalizing and total score [*F*(*p* value)]

	YSR		
	Internalizing	Externalizing	Total
Opt vs. non-opt			
PBI father			
CBCL int	1.17 (.32)	–	–
CBCL ext	–	4.08** (.007)	–
CBCL total	–	–	1.63 (.18)
PBI mother			
CBCL int	0.80 (.50)	–	–
CBCL ext	–	0.91 (.44)	–
CBCL total	–	–	1.05 (.37)
Opt vs. affectionless control			
PBI father			
CBCL int	1.21 (.31)	–	–
CBCL ext	–	2.87* (.037)	–
CBCL total	–	–	1.29 (.28)
PBI mother			
CBCL int	1.71 (.16)	–	–
CBCL ext	–	1.62 (.19)	–
CBCL total	–	–	2.11 (.10)
Opt vs. affectionate constraint			
PBI father			
CBCL int	0.98 (.40)	–	–
CBCL ext	–	1.79 (.15)	–
CBCL total	–	–	0.92 (.43)
PBI mother			
CBCL int	1.14 (.34)	–	–
CBCL ext	–	0.50 (.68)	–
CBCL total	–	–	0.60 (.62)
Opt vs. neglectful			
PBI father			
CBCL int	1.22 (.30)	–	–
CBCL ext	–	3.50* (.017)	–
CBCL total	–	–	1.56 (.20)
PBI mother			
CBCL int	1.10 (.35)	–	–
CBCL ext	–	0.66 (.58)	–
CBCL total	–	–	0.75 (.53)

*** *p* < .001, ** *p* < .01, * *p* < .05

## Discussion

Our data show a high correlation between behavioral and emotional traits in childhood and those in young adulthood, suggesting a general consistency of behavior and emotional profiles/traits over time. This is especially interesting, since parents completed the CBCL of children at 7–9 years of age, while the young adult participants completed the YSR at a mean age of 18 years. This finding also suggests a high inter-rater reliability of these screening instruments over time in our cohort. Second, our data show strong main effects of perceived maternal care and paternal care, suggesting that both are influential for socio-emotional development. Third, although we found evidence of a differential effect of parental care as a function of socio-emotional status in childhood, as reflected in the CBCL data, this effect was only unique to externalizing scores in the YSR. Thus, we report a greater effect of paternal care on externalizing scores among youth with higher externalizing scores in childhood. This finding is consistent with previous reports of increased susceptibility to the effects of parenting among young children with more difficult childhood temperament [25].

Interestingly, while we found the main effects of both perceived maternal and paternal care on socio-emotional function as reflected in the YSR, the interaction effect between externalizing scores in childhood and care on externalizing scores on the YSR was apparent only for paternal care (Table 5; Fig. 1). This interaction effect was significant for optimal versus non-optimal parenting and specifically for affectionless control and neglectful parenting. These findings suggest that optimal parenting by the father is especially important for children who score high on externalizing problems in childhood, although optimal parenting of the mother also seems to decrease externalizing problems over time. For children with elevated externalized traits, optimal parenting by the father seems to be a protective factor; hence, highly reactive or negative children are more susceptible to positive parenting by the father than their less reactive peers. This finding is in concordance with previous findings of increased susceptibility to parenting styles of children with difficult behavioral and emotional traits [25, 32].

Our findings are in agreement with previous studies, which suggested differential effects of caretaking by mothers or fathers. Thus, associations of ADHD with comorbid externalizing problems were negatively associated with care of the father [14]. A study by Narusyte and colleagues [33] found that critical remarks of father, but not mother, were associated with externalizing behavior of adolescents. Other studies also suggest the importance of the paternal role with increasing age of the child. For example, in a sample of 1364 children of ten different Western geographic areas, maternal support for child autonomy in boys was mediated by higher self-reliance at grade one, while the paternal support for child autonomy was associated with increase in self-reliance in boys for a longer period, from grades one to three [34]. In addition, having a caring father has been associated with lower risk of certain psychopathologies, such as posttraumatic stress disorder [35].

In contrast to previous studies, we did not find any interaction effect of perceived parenting styles and socio-emotional status in childhood on later internalizing problems [36]. This inconsistency in findings might be explained by the differences in the study population, study design, stability of internalizing traits in our population, or differences between parenting practices in different cultures. Although some parenting practices (e.g., nurturing and protecting offspring [37]) and parent–child interaction therapies are applicable across cultures [38], a discrepancy has been described between parenting styles in Asia in comparison to European-American cultures [39], with parenting styles in Asia being more controlling than in Europe and America. In contrast to the existing literature, a higher percentage of Singaporean mothers and fathers in our study displayed affectionless control as well as neglectful parenting styles, but a lower percentage displayed affectionate control parenting styles when compared to other non-clinical samples from Europe [40]. Despite the differences in parenting styles, we only found interaction effects between paternal parenting styles, but not between controlling or neglectful parenting styles of the mother with socio-emotional status in childhood on mental health in adolescents when assessed across the entire sample. A possible explanation can be gleaned from previous studies that showed differential effects of maternal parenting styles in Asian cultures as compared to westernized cultures. There are a number of reports showing that controlling styles of mothers associate with positive outcomes in Asian participants. For example, Li and colleagues [41] found that perceived maternal authoritarian parenting styles were related to positive socio-emotional development in Chinese sample, but not in the European young adults.

A major strength of this study is the prospective study design with a reasonable sample size for a longitudinal study conducted over more than 10 years. Additionally, internalizing and externalizing problems were explored over the full range of the spectrum, with most children being in the normal range. Our study also has limitations. First, the mean score on the YSR externalizing scale is in the lower range; hence, most children do not have serious externalizing problems. As development trajectory studies show that a reduction of externalizing problems is expected for both sexes from childhood to adolescence [3], this raises the question on how much of this improvement in externalizing symptoms can be attributed to the parenting style of the father. Although in our study the overall externalizing symptoms indeed decreased over time, we still found an interaction effect with optimal parenting of father suggesting that a perceived optimal parenting style of father further reduces externalizing symptoms from childhood to early adulthood. Second, the data are based on subjective information. However, we found a high correlation between CBCL, filled out by parents, and YSR, filled out by the adolescents, which suggests high inter-rater reliability. Moreover, in previous studies, child reports of parenting have been widely used and found to be consistent with parents’ and observers’ reports [42, 43]. Furthermore, subjective experiences are thought to have a greater impact on physiology and behavior response than objective measurements [44]. Third, in our study we have investigated the parenting styles of mother and father separately, while in “real life” both parenting styles would have interacted and created an impact on the child. Also, a one- or two-parent household may influence the effect of parenting styles on the child. Although it may be difficult to collect this information over a longer period, as this may change over time, it is a limitation that we lack this information in our study. Another drawback is that our findings may not be representative of the whole Singaporean population, as the socioeconomic status in our sample was slightly higher as compared to the national sample and the children had generally a higher IQ. Although we controlled for socioeconomic status, our data may not be representative of parents and children with a very low socioeconomic status.

## Conclusion

Our results suggest that optimal parenting of both parents is essential in social emotional development and an optimal parenting style of the father may moderate externalizing symptoms from pre-puberty to puberty. Future studies should replicate and further explore the role of parenting over an extended time period from childhood into young adulthood. Additionally, it will be important to explore the maternal and paternal parenting styles together. Previous studies found low levels of similarity between maternal and paternal parenting styles. Studies also showed that dissimilarity between parenting styles is an important factor in the development of socio-emotional problems in children and that more similarity in some parenting styles between parents is associated with more compliant behavior in children [45–48]. Understanding the influence of different parenting styles on the child will have long-term benefits, as early intervention programs can help to improve parenting styles and as such may improve health outcomes for children.
